# United States Veterinary Medical Association

**Published:** 1893-11

**Authors:** 


### EDITORIAL.

### THE UNITED STATES VETERINARY MEDICAL ASSOCIATION.

Who would have believed ten years ago, aye, even five years ago, that this association could have held a profitable meeting covering a period of nearly one week. Yet such was the case at Chicago, when five days of constant work, with the exception of half of one day spent in enjoying the hospitality of the Illinois State Society, was not sufficient to finish the consideration of the subjects brought before the association. Some important papers had to be omitted, but it was unanimously voted to have them printed in the proceedings of the association. This was an especially important meeting, from the fact that it was not only the regular annual meeting of our association, but the "The First International Veterinary Congress of America." It is much to be regretted that there were no veterinarians present from abroad, but we may feel certain from the letters of regret presented that it was the time of meeting which prevented them from attending. It is a pity that such was the case, for every veterinarian present could not but have been proud of the association. Never in the history of the association has the attendance been so large or the enthusiasm so great. Many new faces were to be seen, and events proved that we have excellent material in all parts of the country.

We have arrived at a time when the association is truly national in character, and tnat it may ever continue so is the wish of all sincere members of the organization.

Excellent reports were received from the different committees, and splendid administrative work was done. So rapid has been the stride with which this association has advanced during the past few years, that it is hardly possible to estimate the influence for good which it may have upon the profession in the future.

Our recent meetings have demonstrated to the country that ours is a learned profession and entitled to recognition as such. We are now beginning to reap the fruits of our endeavors. Learned men from our sister profession arc pleased to join us in our work, and to lend their influence toward a closer union of the two branches of the medical profession.

As our numbers increase, the work of the association becomes extended, and we surmise that in the near future provision will have to be made for the carrying on of the work of different sections at one and the same time. This will possess the advantage of a method for the more rapid dispatch of work, but it will have the disadvantage of separating the members and a tendency to specialization.

It is a question, then, which the committee on reorganization may have to consider, whether it will be better to divide the meetings up into sections or to curtail the number of papers to be brought before the association. For our part we should prefer the latter for the present. The various committees will have a busy year of it, but we are certain that the appointing power could not be in better hands, and it is to be hoped that those appointed will get to work early and with a will, for upon the work of committees depends, to a great extent, the success of the organization.

A. W. C.

The reports of the proceedings arrived too late to be properly revised for publication in this number of the Journal. They, with some of the papers and committee reports, will appear in the December and following issues. About two hundred (200) veterinarians, representative men from every part of the country, were present during the Congress. The seven (7) sessions had each an average attendance of one hundred and twenty-five (125). Not less than nine (9) representatives of as many Agricultural Experiment Stations assisted at the meetings.

One of the first acts of the Congress was its decision to secure from the National Government an Act of Incorporation; this work has been already placed in the hands of the committee to carry into execution.

As a means of winning away from indiscriminate advertising on letter headings, etc., the association decided to adopt an association emblem, and for this purpose the seal will be the insignia. Two sizes of the same were decided upon ; one about as large as a ten cent piece, and the other a twenty-five cent piece.

The association, on recommendation of the Comitia Minora, declined to recognize the Ohio Veterinary College and the Detroit Veterinary College as at present organized. They also denied recognition to applicants for membership from the New York Veterinary College, during the years of "'92" and "'93," on the grounds that it was not sufficiently well equipped, from a veterinary standpoint, to properly qualify members of the profession for membership in the organization. The closest scrutiny was exercised in the list of applicants for membership, and 20 per cent. of said applicants were declined the privilege of membership in the organization.

Resolutions were adopted, with but one dissenting voice placing the organization on record against the "spoils system" in the selection of veterinarians for the various places where they are recognized under the National Government, and asking that the merit system be established by those having the responsibilities of these selections in charge.

The election for officers showed great judgment in the selection of men fitted to the positions and adapted to the needs of the veterinary organization throughout the country. The choice for President is but a just tribute to the zealous and faithful work of Dr. Hoskins, who has done more than anyone else in bringing the association to its present standard of perfection.

Officers elected for 1894:

*President*—Dr. W. H. Hoskins, 3452-54 Ludlow Street, Philadelphia, Pa.

*Vice-President*—Dr. A. W. Clement, 916 Cathedral Street, Baltimore, Md.

*Treasurer*—Dr. Jas. L. Robertson, 409 Ninth Avenue, New York.

*Secretary*—Dr. T. J Turner, Columbia, Missouri.

